# Antibody response in patients admitted to the hospital with suspected SARS-CoV-2 infection: results from a multicenter study across Spain

**DOI:** 10.1007/s10096-020-04139-5

**Published:** 2021-01-29

**Authors:** Ana Fuentes, Esther Serrano-Conde, Carolina Roldán, Rafael Benito-Ruesca, Gregoria Mejías, Antonio Sampedro, Gabriel March-Roselló, Isabel Fernández-Natal, Juliana Esperalba, Mario José Rodríguez, Paula Martínez de Aguirre, Carlos Salas, María Lourdes Roc, Luis Miguel Soria, Mónica Parra-Grande, María Dolores Montero, Ricardo Fernández-Roblas, Francisco Franco-Álvarez de Luna, Carmen Lozano, Federico García

**Affiliations:** 1 Hospital Universitario Clínico San Cecilio, Instituto de Investigación Ibs, Av, Innovación S/N, 18016 Granada, Spain; 2 Complejo Hospitalario de Jaén, Jaén, Spain; 3 Hospital Clínico Universitario Lozano Blesa, Zaragoza, Spain; 4 Hospital Universitario de Burgos, Burgos, Spain; 5 Hospital Universitario Virgen de las Nieves, Granada, Spain; 6 Hospital Clínico Universitario de Valladolid, Valladolid, Spain; 7 Complejo Asistencial Universitario de León, León, Spain; 8 Hospital Universitario Valle de Hebrón, Barcelona, Spain; 9 Hospital Ramón y Cajal, Madrid, Spain; 10 Clínica Universidad de Navarra, Pamplona, Spain; 11 Hospital Universitario Marqués de Valdecilla, Santander, Spain; 12 Hospital Universitario Miguel Servet, Zaragoza, Spain; 13 Hospital San Pedro, Logroño, Spain; 14 Complejo Hospitalario Universitario de Albacete, Albacete, Spain; 15 Hospital Universitario La Paz, Madrid, Spain; 16 Hospital Universitario Fundación Jiménez Díaz, Madrid, Spain; 17 Hospital Universitario Juan Ramón Jiménez, Huelva, Spain; 18 Hospital Universitario Virgen del Rocío, Sevilla, Spain

**Keywords:** COVID-19, SARS-COV-2, IgG, IgM, IgA, Diagnosis

## Abstract

**Aim:**

To evaluate the serological response against SARS-CoV-2 in a multicenter study representative of the Spanish COVID pandemic.

**Methods:**

IgG and IgM + IgA responses were measured on 1466 samples from 1236 Spanish COVID-19 patients admitted to the hospital, two commercial ELISA kits (Vircell SL, Spain) based on the detection of antibodies against the viral spike protein and nucleoprotein, were used.

**Results:**

Approximately half of the patients presented antibodies (56.8% were IgM + IgA positive and 43.0% were IgG positive) as soon as 2 days after the first positive PCR result. Serological test positivity increased with time from the PCR test, and 10 days after the first PCR result, 91.5% and 88.0% of the patients presented IgM + IgA and IgG antibodies, respectively.

**Conclusion:**

The high values of sensitivity attained in the present study from a relatively early period of time after hospitalization support the use of the evaluated serological assays as supplementary diagnostic tests for the clinical management of COVID-19.

## Introduction

Coronavirus disease 2019 (COVID-19) is a severe acute respiratory syndrome produced by a novel coronavirus (SARS-CoV-2) that has spread globally and very quickly since its first appearance in Wuhan, China, in December 2019 [1]. SARS-CoV-2 is the seventh known coronavirus that infects humans; SARS-CoV, MERS-CoV, and SARS-CoV-2 can cause severe disease, whereas HKU1, NL63, OC43, and 229E are associated with mild respiratory illness [2]. The virus has a genome size of 30 kilobases that encodes multiple structural and nonstructural proteins. The structural proteins include the spike (S) protein, the envelope (E) protein, the membrane (M) protein, and the nucleocapsid (N) protein.

The diagnostic approach to SARS-CoV-2 includes the detection of viral RNA by real-time PCR (RT-PCR). Different factors could contribute to false negative results of RNA tests with RT-PCR, such as insufficient amount of virus at the site of sample collection, incorrect sample collection or being outside in the viral replication time window [3–6]. Serological methods combined with PCR could be of help for increasing the sensitivity and accuracy of the diagnosis, especially in patients with negative RT-PCR results; serology may also help to identify asymptomatic and past infections. SARS-COV-2 serology undoubtedly helps to understand the immune status of the population and to evaluate viral spread [7]; hence, serology should be used for epidemiological studies to investigate the rate of asymptomatic infections and to better estimate morbidity and mortality [7]. Serological methods include binding and neutralization assays. Binding assays such as ELISAs are easily automatized, and they are very well adapted to a pandemic situation; neutralization assays require viral culture, and they must be performed in a facilities with higher biosecurity levels [8].

Preliminary studies have analyzed antibody responses against SARS-COV-2. Some authors [2, 9–12] found that IgM was detected on day 7 and peaked on day 28, and IgG appeared by day 10 and peaked on day 49, while others [13] determined that seroconversion among 173 patients took place at median times of 12 (IgM), 14 (IgG), and 11 (neutralizing antibodies) days. The duration and nature of SARS-CoV-2 immunity is unknown. The timescale of protection is a critical determinant of the future impact of the pathogen. The presence or absence of protective immunity due to infection or vaccination (when available) will affect future transmission and illness severity and will allow the identification of individuals with protective immunity [2, 7, 10, 13, 14].

Additional studies are needed to characterize how anti-SARS-CoV-2 antibodies will change over prolonged periods of time. The present study presents data from a large series of samples covering both IgG and IgM + IgA responses to two of the main viral antigenic proteins (N and S).

## Materials and methods

### Patients

One thousand two hundred thirty-six patients screened for COVID-19 admitted to 18 Public and Private Spanish Hospitals (Clínica Universidad de Navarra, Complejo Asistencial Universitario de León, Complejo Hospitalario Universitario de Albacete, Complejo Hospitalario de Jaén, Hospital Clínico Universitario Lozano Blesa, Hospital Clínico Universitario de Valladolid, Hospital Ramón y Cajal, Hospital San Pedro, Hospital Universitario Clínico San Cecilio, Hospital Universitario de Burgos, Hospital Universitario Fundación Jiménez Díaz, Hospital Universitario Juan Ramón Jiménez, Hospital Universitario La Paz, Hospital Universitario Marqués de Valdecilla, Hospital Universitario Miguel Servet, Hospital Universitario Valle de Hebrón, Hospital Universitario Virgen de las Nieves, and Hospital Universitario Virgen del Rocío) were studied. All patients were positive by RT-PCR; sex data were available for 513 men and 353 women; age data were available for 1066 patients, mean age 64 years, range 15–100 years.

### Serum samples

Samples were drawn on the same day or after the RT-PCR test was performed. Differences in days were due to clinical needs in the general management of the patients.

Single serum samples were obtained from 1054 patients, while multiple serum samples (*n* = 413) were obtained from 183 patients, comprising 1467 samples. The distribution of samples according to time from RT-PCR can be seen in Table 1. There were two samples available for 43 patients, the first collected before 4 days after PCR and the second collected 7–17 days after PCR.

**Table 1:** Positive rates for IgG and IgA + IgM detection in 1466 serum samples from PCR-positive patients. The results are expressed as absolute frequencies (percentage shown in parentheses) of positive samples in each parameter or in both parameters at the same time

Sample day^a^	Total no. of sera	IgG	IgM + IgA	IgG and/or IgM + IgA	Only IgG positive	Only IgM + IgA positive	Both IgG and IgM + IgA positive	Both IgG and IgM + IgA negative
0	326	135 (41.4)	154 (47.2)	187 (57.4)	19 (5.8)	38 (11.7)	116 (35.6)	153 (46.9)
1	332	134 (40.4)	165 (49.7)^b^	180 (54.2)	21 (6.3)	52 (15.7)	113 (34.0)	146 (44.0)
2	154	83 (53.9)	98 (63.6)	95 (61.7)	6 (3.9)	21 (13.6)	77 (50.0)	50 (32.5)
3	106	61 (57.5)	68 (64.2)	69 (65.1)	8 (7.5)	15 (14.2)	53 (50.0)	30 (28.3)
4	111	79 (71.2)	89 (80.2)	92 (82.9)	7 (6.3)	17 (15.3)	72 (64.9)	15 (13.5)
5	51	40 (78.4)	42 (82.4)	39 (76.5)	2 (3.9)	4 (7.8)	38 (74.5)	7 (13.7)
6	53	34 (64.2)	38 (71.7)	38 (71.7)	1 (1.9)	5 (9.4)	33 (62.3)	14 (26.4)
7	50	37 (74.0)	42 (84.0)	41 (82.0)	1 (2.0)	6 (12.0)	36 (72.0)	7 (14.0)
8	42	26 (61.9)	33 (78.6)	32 (76.2)	1 (2.4)	8 (19.0)	25 (59.5)	8 (19.0)
9	30	20 (66.7)	23 (76.7)	23 (76.7)	1 (3.3)	4 (13.3)	19 (63.3)	6 (20.0)
10–16	129	114 (88.4)	119 (92.2)	118 (91.5)	2 (1.6)	7 (5.4)	112 (86.8)	8 (6.2)
17–23	40	36 (90.0)	36 (90.0)	38 (95.0)	1 (2.5)	1 (2.5)	35 (87.5)	3 (7.5)
24–30	16	16 (100)	16 (100)	16 (100)	0 (0.0)	0 (0.0)	16 (100)	0 (0.0)
> 30	26	24 (92.3)	20 (76.9)	24 (92.3)	4 (15.4)	0 (0.0)	20 (76.9)	2 (7.7)

^a^Days after the first positive PCR result

^b^*p* = 0.016 compared to IgG (all other comparisons not significative)

### ELISAs

Anti-SARS-CoV-2 IgG and IgM + IgA ELISAs (Vircell SL, Spain) were carried out according to the manufacturer’s protocol. Reaction wells in both assays were coated with nucleocapsid and spike proteins. Serum samples were previously inactivated at 56 °C for 30 minutes. Samples were immediately tested after inactivation or stored at 4 °C for no longer than 4 days before testing. The specificity declared by the manufacturer for the IgG and IgM + IgA assays is 98.2% and 98.9%, respectively, based on studies performed on prepandemic populations. Both ELISAs are qualitative; the IgM + IgA ELISA does not differentiate between both inmunoglobulins.

### RT-PCR assays

RT-PCR from naso- and oro-pharyngeal swabs was performed after nucleic acid extraction with different commercial CE-approved assays.

### Statistical analysis

Statistical analysis was performed with the help of the R program version 3.6.3 (2020-02-29)—“Holding the Windsock” Copyright (C) 2020 The R Foundation for Statistical Computing Platform: x86_64-w64-mingw32/x64 (64-bit). Borderline results were primarily interpreted as negative. The Wilcoxon-Mann-Whitney test was used for p-value calculations. Data were graphically presented in box-and-whisker plots with boxes encompassing 90% of the data and whiskers presenting the 95% and 5% percentiles.

## Results

### Anti-SARS-CoV-2 antibody kinetics

The results from 1467 serum samples from PCR-positive patients referred to the collection date with respect to the first PCR-positive result of the patient are shown in Table 1. At day 0, a higher reactivity was observed for IgM + IgA (154 positive samples, 47.2%) than for IgG (135, 41.4%), with 187 samples (57.4%) with any serological marker. This tendency for a higher reactivity in the IgM + IgA response only reached statistical significance at day 1 (*p* = 0.016), and could be seen during the first 16 days, with 84.0% of IgM + IgA positive results against 74.0% of IgG positive results at day 7 after PCR. After the third week, the proportion of positive results was similar in both parameters, while IgG was more prevalent in samples collected one month after PCR. Positive IgM + IgA together with positive IgG was the most frequently found pattern (36.5%) at day 0, followed by positivity to only IgM + IgA (11.7%) and positivity to only IgG (5.8%). Throughout all the time ranges studied and whenever a single marker was present, IgM + IgA was more frequent than IgG.

**Fig. 1:**
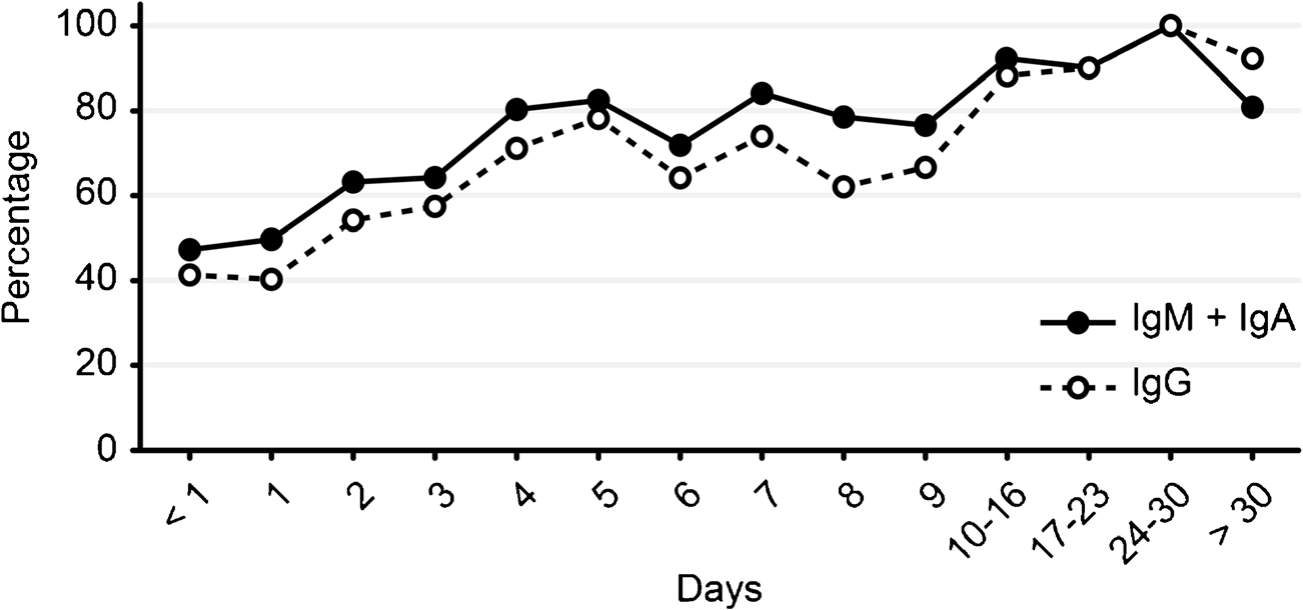
Dynamic trend of the positive rate for IgG and IgM + IgA in sera of RT-PCR-positive patients

Figure 1 shows the evolution of IgG and IgM + IgA over time. The proportion of positive cases was higher for IgM + IgA until day 24, when positivity for both IgG and IgM was equal.

To better understand the kinetics of the antibody response, seroconversion was studied in 43 patients for whom two samples were available (T1: samples collected not later than 4 days post PCR; T2, second samples collected between 7 and 17 days post PCR). The results are shown in Table 2. IgG was detected in 15 (34.9%) of the T1 samples, while IgM + IgA was detected in 23 (53.5%). IgG and IgM + IgA were detected in 35 (81.4%) and 40 (93.0%) of the T2 samples, respectively. Twenty patients seroconverted for IgG and 19 for IgM + IgA, whereas 2 patients did not show seroconversion. The index means were 1.97 (IgG) and 2.55 (IgM + IgA) for the acute infection samples and 3.04 (IgG) and 3.15 (IgM + IgA) for the convalescent samples.

**Table 2:** Positive rates for IgG and IgA + IgM detection in sera from selected patients for whom two samples collected at two different time points were available. Time ranges were 0–4 days post PCR for first serum collection and 7–17 days post PCR for second serum collection

Serological parameter	No. of positive sera (%)	
	0–4 days	7–17 days
IgG	15 (34.9)	35 (81.4)
IgM + IgA	23 (53.5)	40 (93.0)
Both IgG and IgM + IgA	15 (34.9)	34 (79.1)
IgG only	1 (2.3)	1 (2.3)
IgM + IgA only	8 (18.6)	6 (14.0)
Both negative	19 (44.2)	2 (4.7)

*T1* first serum, *T2* second serum

### Effect of age and sex on antibody responses to SARS-CoV-2

**Fig. 2:**
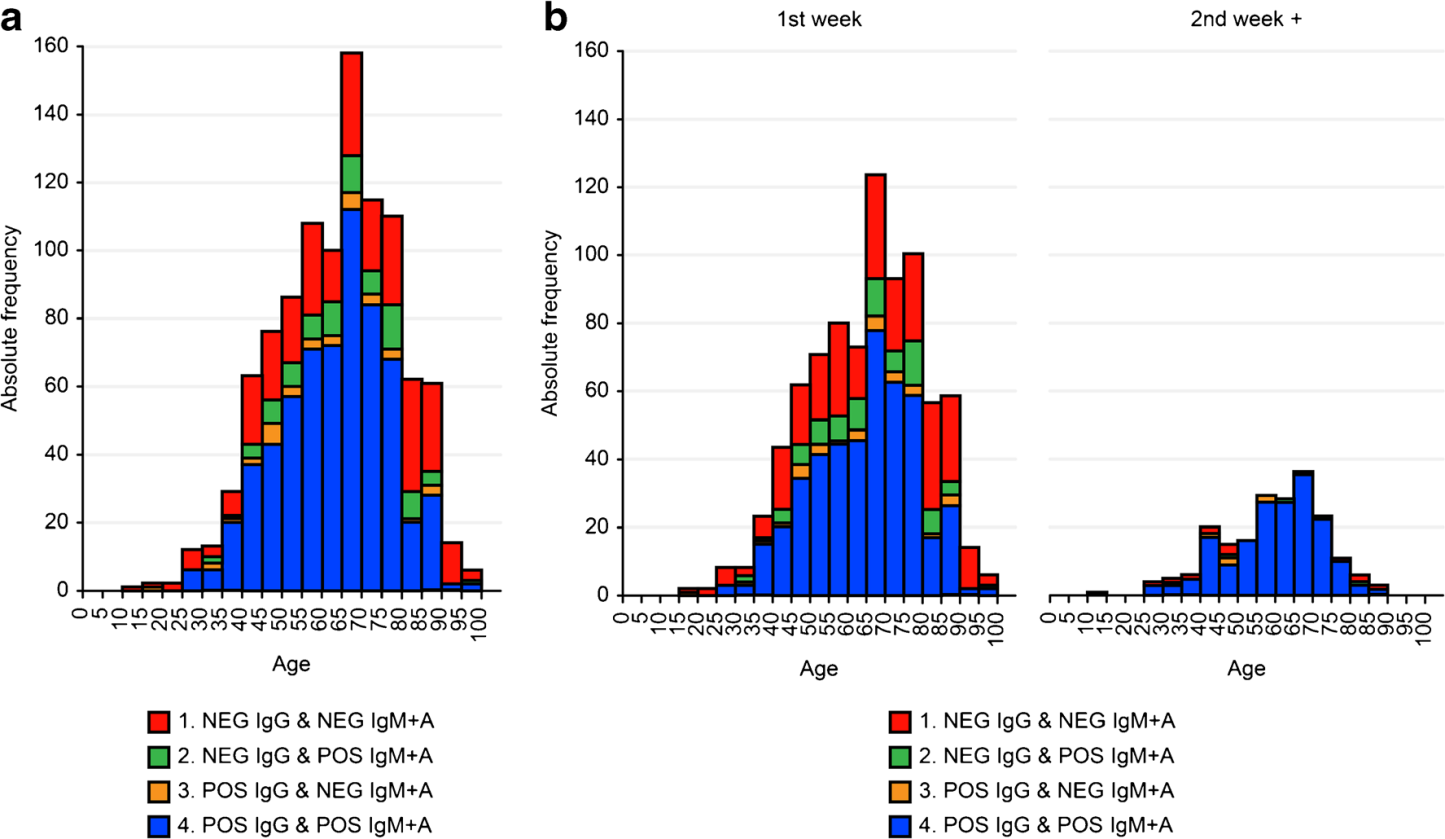
Serology test results for positive PCR patients grouped by age. Bar heights represent absolute frequencies. **a** Data from all PCR-positive patients, **b** results corresponding to samples collected in the first week after the first PCR result, and **c** results corresponding to samples collected later than one week after the first PCR result

PCR-positive patients were classified into four groups according to the results of the serological tests: only IgG positive, only IgM + IgA positive, positive in both tests or negative in both tests. Figure 2a shows the distribution of serological results by age group corresponding to the 1066 patients for whom age data were available. The proportion of patients with negative serological responses for both tests increased with patient age. When these results were analyzed according to the time elapsed between the PCR and the serum collection (Fig. 2b and c), this effect could also be seen for samples collected early after the first PCR diagnosis, while most samples collected in the second week after PCR showed positive serological responses in all age groups.

**Fig. 3:**
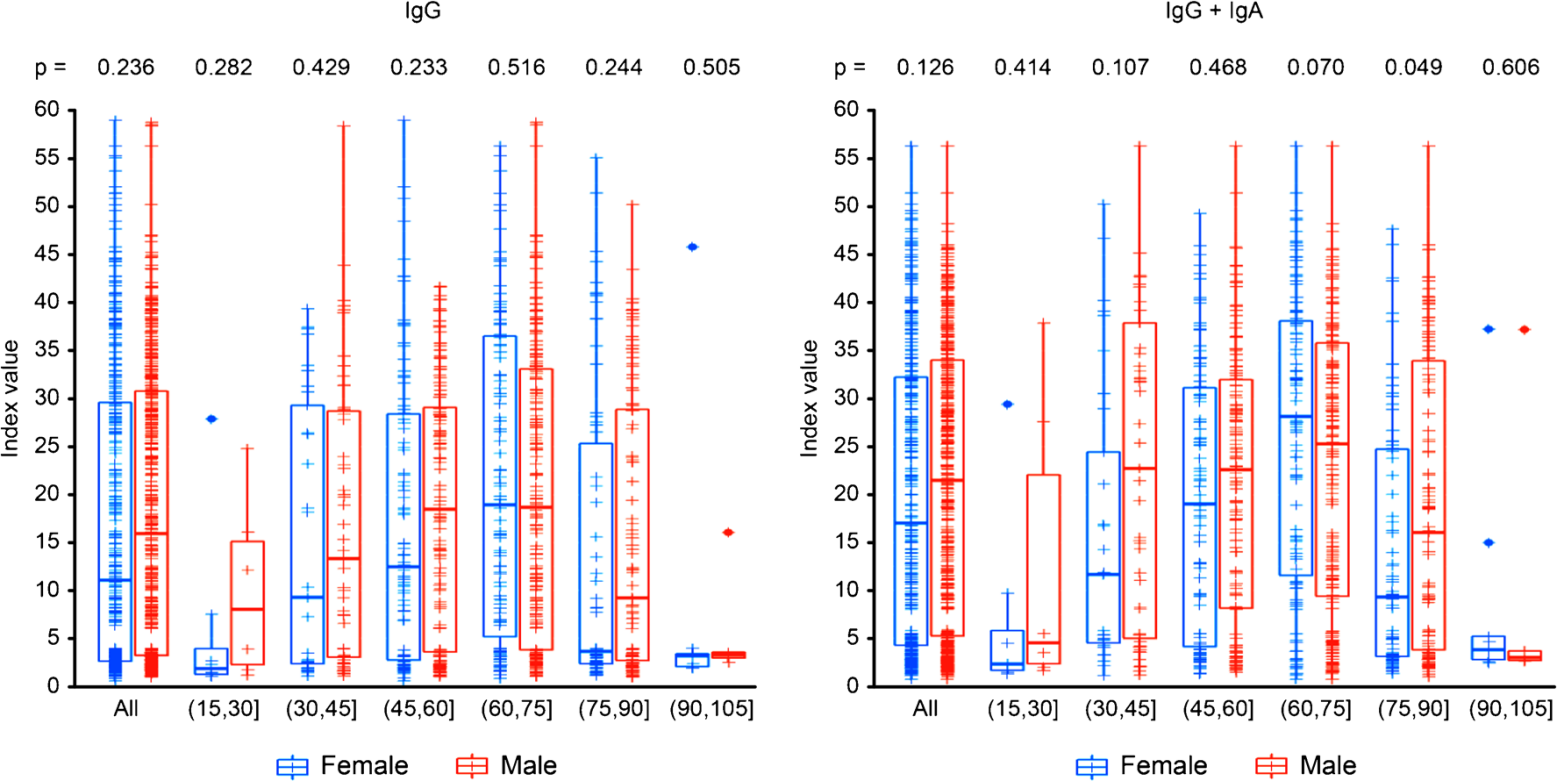
IgG and IgM + IgA index value (10 X serum/cutoff) boxplot for all PCR-positive patients distinguishing between male and female and grouped by age. Boxes encompass 90% of the data, and whiskers present the 95% and 5% percentiles

Figure 3 shows the differences in serological results by age and sex. No significant differences were seen between men and women as a whole either for IgG or for IgM + IgA. Although differences between the sexes could be observed for some age groups, there was not a clear tendency that supported a higher serological response of men over women, and this result may reflect differences in the population sizes.

## Discussion

The utility of serological tests to help in the diagnosis of COVID-19 was evaluated in a multicenter study with a large series of samples collected from 1236 patients in several Spanish hospitals during the peak of the pandemic. In our study, we observed high values of sensitivity, especially for the IgM + IgA test at early periods of time after hospitalization (61.7% presented a positive serology in the first 4 days of the diagnosis, and 90.5% after the first week). We believe that our findings support the use of serological assays as supplementary diagnostic tests for the clinical management of COVID-19. We also observed a decreased sensitivity of serology in older patients in samples collected early in the disease.

Two commercial kits were used to measure the IgG and IgM + IgA responses against two major antigenic components of the virus, the N and S proteins. To our knowledge, few commercial tests are simultaneously based on both proteins. Studies with good performances for assays based either on the N protein [15, 16], the S protein [7, 13, 17] or both antigens [9, 10, 14, 18] have been published. Another peculiarity of the assays used in the study is the inclusion of IgA together with IgM for early detection of the disease. Several studies have described a good sensitivity of this immunoglobulin class, better than that of IgG, although with a lower specificity [2, 7, 12, 18, 19]. The specificity of the test was established by the manufacturer of the kit on the basis of samples collected from prepandemic populations (98.8% CI 95% 95.52–99.68). The high levels of sensitivity reported in the present study, above those reported in other studies [17, 20, 21], may be related to the aforementioned special features of the assays used in the study: the use of two viral antigens and the inclusion of IgA.

The percentage of IgM + IgA-positive patients with IgG-negative levels was higher in samples collected at early stages of the disease, as can be expected for these immunoglobulin classes. However, we did not observe a decrease in IgM + IgA values, as has been shown by other authors [9], probably due to the low number of samples after hospital discharge included in the study. Positivity of the serological tests increases with time, reaching values close to 100% in samples taken 10 days or more after the first PCR result. Conversely, in studies carried out with MERS patients, 20% of the patients did not show a detectable serological response after more than 30 days of evolution [22]. For SARS, all patients developed antibodies [23], but 28% showed negative IgM after 60 days [24], and most patients had lost the antibody response by six years [25].

However, it is surprising that the large proportion of patients with positive IgG levels in samples were surveyed so close to the first PCR-positive results: 45.0% within the first 3 days. Some of the patients even debuted with an IgG-positive/IgM + IgA-negative pattern. This finding could be in agreement with previous contact with other coronaviruses showing antigenic similarities with the novel SARS-CoV-2, as has been found by authors studying immune cell responses [26], and it could also be explained by the fact that patients are attending the emergency room and need hospitalization, suggesting advanced disease. However, it is not in agreement with the very low levels of IgG against the spike protein and nucleoprotein found in the prepandemic population that translates to the very high specificity of the serological assays used in this and other studies [27].

Due to the large series of sera studied, serological results could be stratified according to the age of the patients. A decreased number of patients with positive serology was observed among the older patients for samples collected early in the disease. However, the proportion increased to near 100% positivity in all age groups for samples collected in the second week or later after the first positive PCR result. Some authors have suggested a relationship between this delayed response in elderly individuals, with less favorable disease evolution in this population group [28]. For the comparison of serological responses between men and women, no global significant differences were observed either in the proportion of positive results or in the immunoglobulin levels attained in each group. The low number of samples in some of the subgroups may have limited the statistical potency of the comparisons.

In summary, the high values of sensitivity attained in the present study from a relatively early period of time after hospitalization support the use of the evaluated serological assays as supplementary diagnostic tests for the clinical management of COVID-19. The large number of samples gives a particular strength to the evaluation compared with that of others previously published. However, the lack of clinical and evolutionary data of the patients, as well as having used time from positive PCR result rather than time from symptom onset, constitutes its major limitations.

## Data Availability

Data may be provided upon request to the corresponding author.
